# A new treatment of hypertrophic and keloid scars with combined triamcinolone and verapamil: a retrospective study

**DOI:** 10.1007/s00238-017-1322-y

**Published:** 2017-06-08

**Authors:** S. B. Kant, E. van den Kerckhove, C. Colla, S. Tuinder, R.R.W.J. van der Hulst, A.A. Piatkowski de Grzymala

**Affiliations:** 1 Department of Plastic Surgery, Maastricht University Medical Center, P Debyelaan 25, 6229HX Maastricht, The Netherlands; 2 KU Leuven, Department of Rehabilitation Sciences, Faber, Universitaire Ziekenhuizen Leuven, Leuven, Belgium; 3 Department of Physical Medicine and Rehabilitation and Burns Center, Universitaire Ziekenhuizen Leuven, Leuven, Belgium

**Keywords:** Hypertrophic scars, Keloids, Kenacort, Verapamil

## Abstract

**Background:**

Since the management of keloid and hypertrophic scars still remains a difficult clinical problem, there is need for adequate, effective therapy. In this study, we explored for the first time the efficacy and the potential synergetic effect of combined triamcinolone and verapamil for the treatment of hypertrophic and keloid scars. The objective was to assess the efficacy of combined intralesional triamcinolone and verapamil therapy for hypertrophic and keloid scars.

**Methods:**

Fifty-eight patients with hypertrophic scars (*n* = 31) and keloid scars (*n* = 27) were included. A specific injection therapy scheme was applied. Five follow-up moments were chosen, with a maximum follow-up of nearly 2 years. The effects of combination therapy on scar pliability, thickness, relief, vascularization, surface area, pain, and pruritus were examined by means of the Patient and Observer Scar Assessment Scale (POSAS).

**Results:**

Our results reveal a fast and abiding improvement of both keloid and hypertrophic scars after treatment with the combination therapy. All POSAS components showed a reduction in scar score, while scar relief, pain, itchiness, and surface area improved significantly (*P* < 0.05) in keloids. Significant improvement in hypertrophic scars was found in scar pigmentation, vascularization, pliability, thickness, pain, and surface area. Overall POSAS scores revealed statistically significant decreases between baseline and 3–4 months, 4–6 months, and >12 months after start of therapy in both keloids and hypertrophic scars.

**Conclusions:**

This study reveals that combined therapy of triamcinolone and verapamil results in overall significant scar improvement with a long-term stable result.

Level of evidence: Level IV, therapeutic study.

## Introduction

Keloids and hypertrophic scars are still a therapeutic problem. These scars are mostly disfiguring and are likely to cause severe psychological problems. Besides the psychological aspect, the physical and functional implications of keloids and hypertrophic scars often cause a notable burden for the patient [1].

The management of hypertrophic scars and keloids remains an unsolved problem. Many therapeutic modalities have been described: intralesional therapy, pressure therapy, cryotherapy, radiotherapy, surgical excision, and even combinations of the earlier mentioned therapies [2–6]. This article focuses on the possibilities that intralesional injections can bring into the therapy of keloids and hypertrophic scars.

The anti-inflammatory and scar-enhancing properties of corticosteroids on hypertrophic scars and keloids have been investigated and documented thoroughly. They are considered a first-line strategy in the treatment of limited keloidal and hypertrophic scars. The most commonly used corticosteroid in this matter is triamcinolone acetonide, and its efficacy and usefulness as well as its limitations are well known [7, 8].

In contrast to corticosteroids, the efficacy of verapamil (a calcium antagonist) and the combination of verapamil and triamcinolone on hypertrophic scars and keloids is less studied. The beneficial effects of verapamil on hypertrophic scars and keloids are mainly addressed as empirically.

Verapamil appears to degrade extracellular matrix by inhibition of collagen production [9, 10]. Furthermore, verapamil may prevent platelet aggregation and decrease neutrophil activity and thereby inhibit inflammation [11].

The Maastricht University Medical Center offers an outpatient clinic exclusively focused on scar treatment and management. With the use of a specific injection regime, we reckon that combination therapy is likely to result in significant scar improvement over time in everyday practice. We believe the positive properties of triamcinolone and verapamil can have a synergetic enhancing effect on hypertrophic scars and keloids when used as combined intralesional therapy. Significant clinical evidence for effectiveness of combined intralesional therapy of triamcinolone and verapamil on hypertrophic scars and keloids in vivo is still lacking.

The aim of this study is to assess the efficacy of combined intralesional therapy of triamcinolone and verapamil in small bothersome hypertrophic and keloid scars.

## Methods

### Design

In this retrospective study, conducted at the department of plastic surgery at the Maastricht University hospital (MUMC+), between July 2012 and December 2015, 58 patients underwent a combined therapy of triamcinolone and verapamil injections in order to improve their hypertrophic or keloid scar. The study includes 58 patients with involvement of in total 31 keloid scars and 27 hypertrophic scars.

### Patients and treated sites

Eligible patients were men or women with keloid or hypertrophic scars, who had not been treated with triamcinolone and verapamil in an earlier stage of their scarring. All patients that received triamcinolone and verapamil treatment in order to improve their scar between July 2012 and December 2015 were included. Major exclusion criteria were the use of an additional scar treatment like pressure therapy or silicone sheets at the time the study started.

The scars of 28 patients had not been treated when the study started. From the remaining 30 patients, 8 of them had been treated solely with ointment and 10 patients were treated with combined silicone and pressure therapy. Other scar therapies, patients previously had included laser therapy, cryotherapy, physiotherapy, silicones, and pressure therapy separately and excision of the scar. Abovementioned therapies were all deemed unsuccessful, and additionally, those treatments took place in a distant earlier stage, causing no interference with the current study. Scar location and scar etiology are documented in Tables 1 and 2, respectively. The study conformed to good clinical practice guidelines and followed the recommendations of the Declaration of Helsinki. The protocol was approved by the local ethics committee.

**Table 1:** Patient characteristics

	Sex		Mean age		Scar location						
	Male	Female	Years	Extremities	Face/head/neck	Pre-sternal	Shoulder	Sternum	Thorax	Abdomen	Back
No.	25	33	28.1 (9–82)	8	18	4	4	13	3	4	4

**Table 2:** Scar etiology and time scars were present when therapy started

	Etiology						
	Acne	Burns	Piercing	Spontaneous	Surgery	Trauma	Varicella
No.	5	1	4	3	33	11	1
	Mean time the scars were present at time therapy started (years)						
	3.84						

### Patients

The relevant patient group after exclusion consisted of 25 men and 33 women with a mean age of 28 years (9–82 years, Table 1) and a mean follow-up of 209 days (39–729 days, Table 3). All patients were diagnosed with hypertrophic or keloid scarring at the scar clinic by a team of experts, consisting of a senior plastic surgeon, a resident plastic surgeon, a prosthetist, and a physiotherapist specialized in scar therapy. Scars were present a mean time of 3.84 years when treatment started (Table 2).

**Table 3:** Follow-up information

	No. of patients	Time after start of therapy (days)	
		Mean	SD
Baseline	58	0	0
1–3 months	17	59.88	15.20
3–4 months	10	103.80	8.52
4–6 months	11	168.45	16.88
6–12 months	11	269.09	58.03
>12 months	9	502.67	108.98
Follow-up time	Days		
Min	39		
Max	729		
Mean	209		

### Procedures

From July 2012 to December 2015, 58 eligible patients were assigned to triamcinolone and verapamil injections that consist of a 1:1 mixture of triamcinolone (Kenacort-A, Bristol-Myers Squibb, New York, United States 40 mg/mL) and verapamil (2.5 mg/mL). The mean volume of the mixture injected in scars was between 0.1 and 0.2 mL.

All patients followed the same injection scheme: a first injection (*t* = 0), the second injection a week after the initial injection, and an additional third injection 3 weeks after the first injection. As from 39 days after the first injection, scars were assessed at the scar clinic by the team of experts.

### Adverse effects

During the study a small amount of patients experienced adverse effects. One patient experienced hardening of the scar. Another patient encountered minor indentation of the scar. Furthermore, a couple of patients experienced a short period of itchiness at the scar directly after the injection.

### Follow-up

In total, 58 eligible patients completely followed the proposed injection scheme as they form baseline. Patients were followed as from 39 to a maximum of 729 days after start of the injection scheme (Table 3).

Based on duration of follow-up, five follow-up moments after baseline (*t* = 0, end of the injection scheme, *n* = 58) were chosen. Follow-up moments consisted of 1 to 3 months (*n* = 17), 3 to 4 months (*n* = 10), 4 to 6 months (*n* = 11), 6 to 12 months (*n* = 11), and >12 months (*n* = 9).

Twelve patients were lost to follow-up because they went to an affiliated hospital for further follow-up, because the recruitment period of the study ended before patients were called for follow-up visit or because patients did not show up for follow-up visit.

### Assessment of the scars

All the scars were evaluated prior to or on the day of the first injection by the previously validated Patient and Observer Scar Assessment Scale (POSAS) [12]. The scar was rated numerically on a ten-step scale by both the patient and doctor on six items: vascularity, pigmentation, thickness, relief, pliability, and surface area on the Observer Scale. The Patient Scale consists of pain, itchiness, color, stiffness, thickness, and irregularity of the scar.

One of the reasons POSAS was chosen for scar evaluation is because it is the only scar assessment tool to include a component for patients to fill in. Furthermore, we chose POSAS because of its distinctive feature of reflecting subjective symptoms like pain and pruritus and because of its appropriateness for everyday practice [13–15].

On each visit, an expert and the patient independently filled out a POSAS form in order to assess the scar.

### Statistical analysis

The study was planned as a case-series study to evaluate the efficacy of triamcinolone and verapamil with respect to scar outcome. Scar scores at follow-up visits are presented as means with standard deviations. Those scores were compared with the use of ANOVA and Games-Howell post-hoc tests for significance in means. A value of *P* < 0.05 was considered statistically significant. Statistical analyses were performed using SPSS version 22.0.0.0.

## Results

### Outcome POSAS scores

The means and standard deviations for baseline and five follow-up moments are presented in Table 4. A one-way ANOVA was conducted to compare mean POSAS scores at baseline and five follow-up moments for keloid scars and hypertrophic scars separately. Post-hoc analyses using the Games-Howell post-hoc criterion were used to make comparisons between follow-up moments. This test was used because it does not assume equal variances and equal group sizes.

**Table 4:** Mean Patient, Observer and POSAS scores for keloids and hypertrophic scars

	Patient score		Observer score		POSAS score	
	Keloids	Hypertrophic scars	Keloids	Hypertrophic scars	Keloids	Hypertrophic scars
Baseline (*t* = 0)	40.73	43.93	27.03	26.67	*67.77*	*70.59*
SD	7.10	6,31	8.22	7.72	10.20	8.79
95% CI	38.08–43.38	41.43–46.42	23.96–30,10	23.61–29,72	63.96–71.58	67.12–74.07
1–3 months	29.90	35.14	21.80	21.57	*51.70*	*56.71*
SD	14.22	8.61	8.14	6.00	20.16	13.51
95% CI	19.73–40.07	27.18–43.11	15.98–27.62	16.03–27.12	37.28–66.12	44.22–69.21
3–4 months	28.57	21.33	18.00	22.00	*46.57*	*43.33*
SD	11.06	8.39	7.64	6.08	12.42	14.43
95% CI	18.34–38.80	0.50–42.17	10.94–25.06	6.89–37.11	35.08–58.06	7.48–79.19
4–6 months	28.50	29.00	20.00	19.80	*48.50*	*48.80*
SD	12.28	6.82	7.69	4.60	11.15	4.97
95% CI	15.62–41.38	20.53–37.47	11.93–28.07	14.08–25.52	36.80–60.20	42.63–54.97
6–12 months	28.80	34.83	17.40	14.17	*46.20*	*49.00*
SD	14,62	13.29	4.16	2.99	17.71	12.67
95% CI	10.65–46.95	20.89–48.78	12.24–22.56	11.02–17.31	24.21–68.19	35.71–62.29
>12 months	23.67	28.17	15.33	18.67	*39.00*	*46.83*
SD	3.79	11.99	2.31	7.69	6.08	14.63
95% CI	14.26–33.07	15.58–40.75	9.60–21.07	10.60–26.73	23.89–54.11	31.48–62.19

### Keloids

**Fig. 1:**
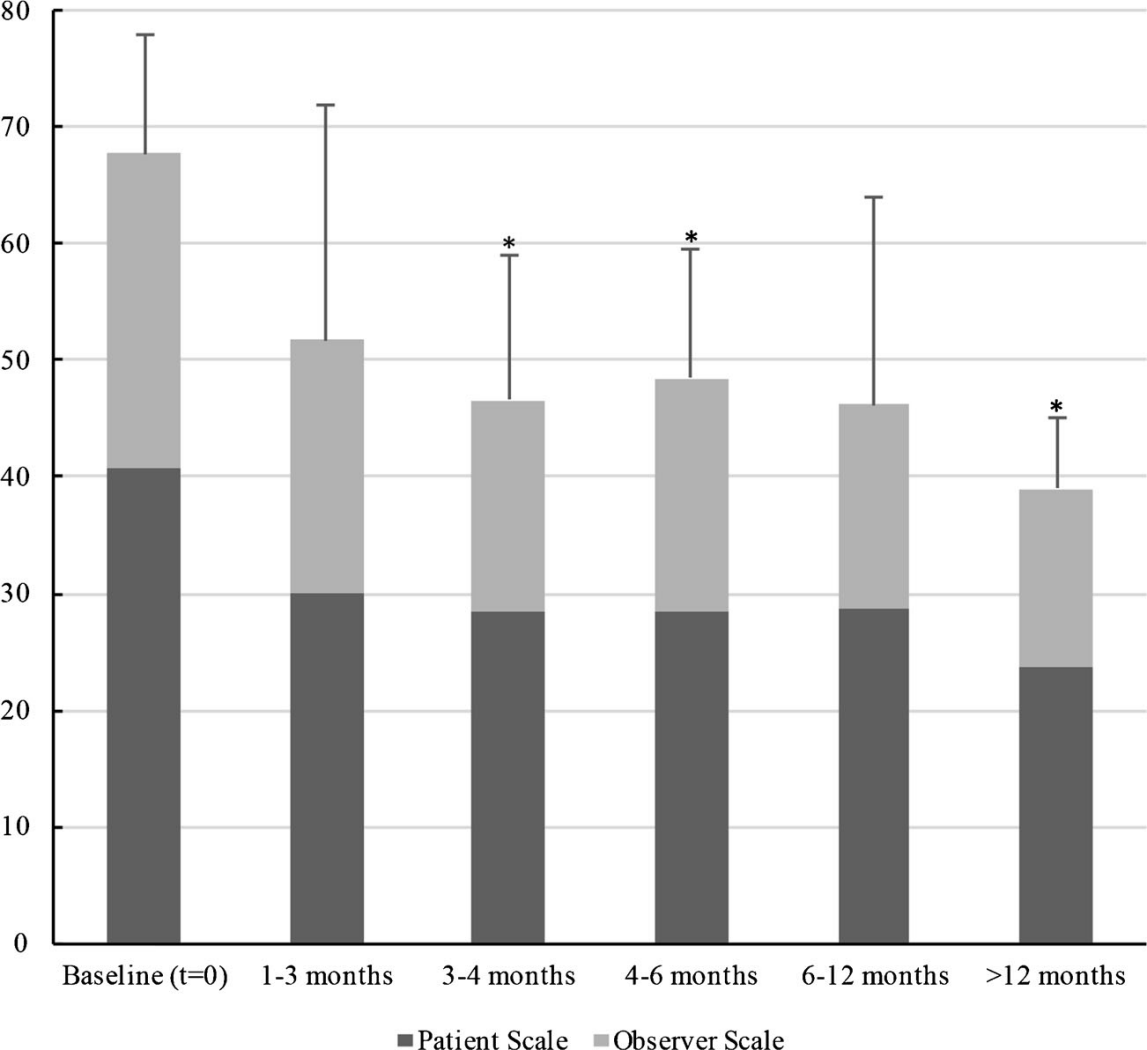
Mean Patient, Observer, and total POSAS scores are shown at baseline and four subgroup visits (early, medium, long, and late term) for keloid scars. A *single asterisk* indicates a statistical significant (*P* < 0.05) difference compared to baseline

For keloids, there were statistical significant differences (*P* < 0.05) in POSAS scores between baseline (67.77, SD: 10.20) and subsequent times (3–4 months (46.57, SD: 12.42), 4–6 months (48.50, SD: 11.15), and >12 months (39.00, SD: 12.59)) (Fig. 1). No statistical significant differences in subsequent times were found. Details about patient, observer, and total POSAS scores at different follow-up moments, standard deviations, and 95% confidence intervals are shown in Table 4.

### Hypertrophic scars

**Fig. 2:**
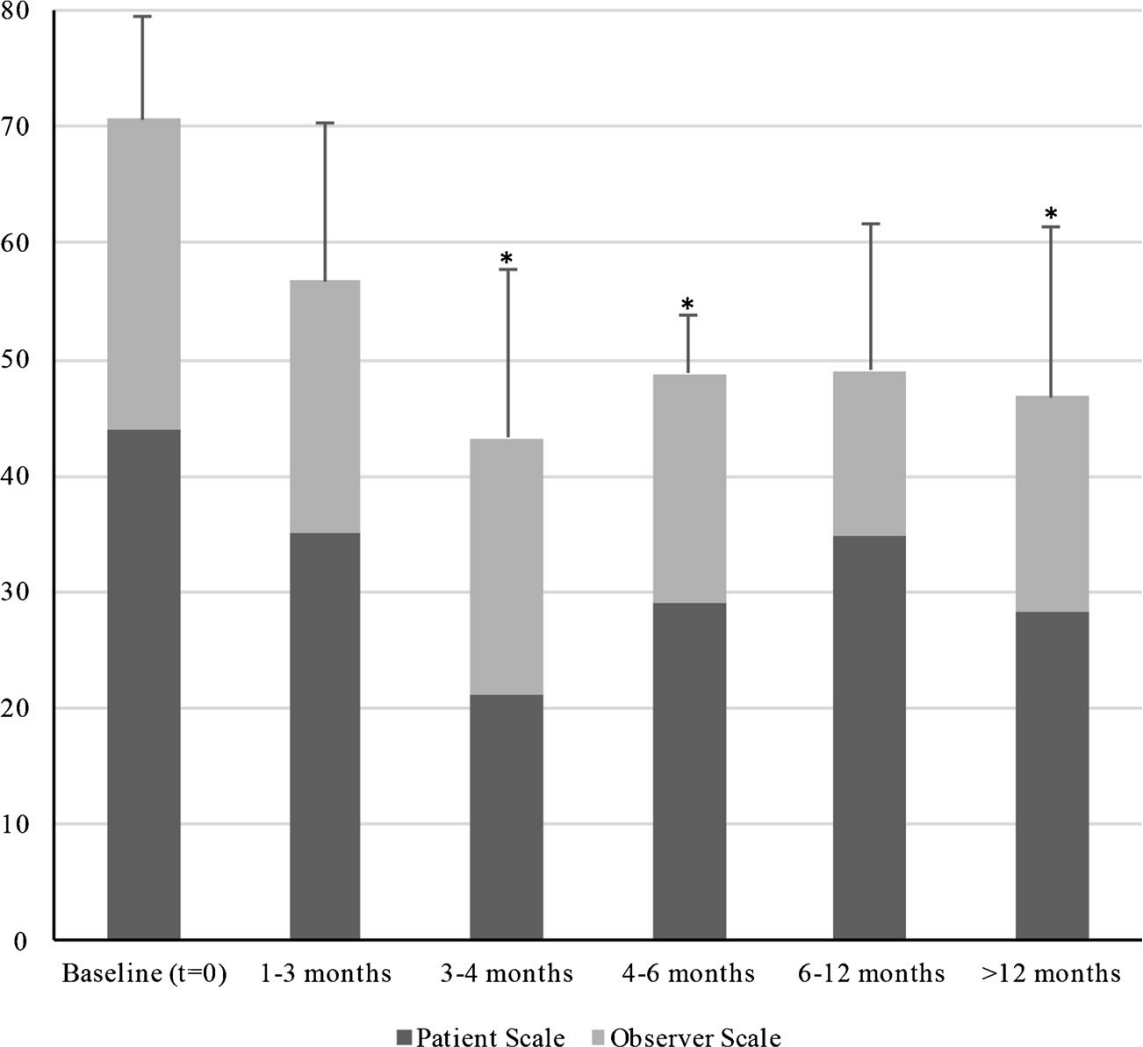
Mean Patient, Observer, and total POSAS scores are shown at baseline and four subgroup visits (early, medium, long, and late term) for hypertrophic scars. A *single asterisk* indicates a statistical significant (*P* < 0.05) difference compared to baseline

For hypertrophic scars, significant improvement in POSAS scores was found between baseline (70.59, SD: 8.79) and subsequent times (3–4 months (43.33, SD: 14.43), 4–6 months (48.80, SD: 4.97), and >12 months (46.83, SD: 14.63)) (Fig. 2, Table 4). Also, no statistical significant differences in subsequent times were observed.

### Patient Scores

To evaluate the outcome of the patient component of the POSAS (pain, itchiness, pigmentation, pliability, thickness, and relief) all Patient Scores were compared on baseline and five follow-up moments. A one-way ANOVA with analyses using Games-Howell post-hoc test was used.

### Keloids

**Fig. 3:**
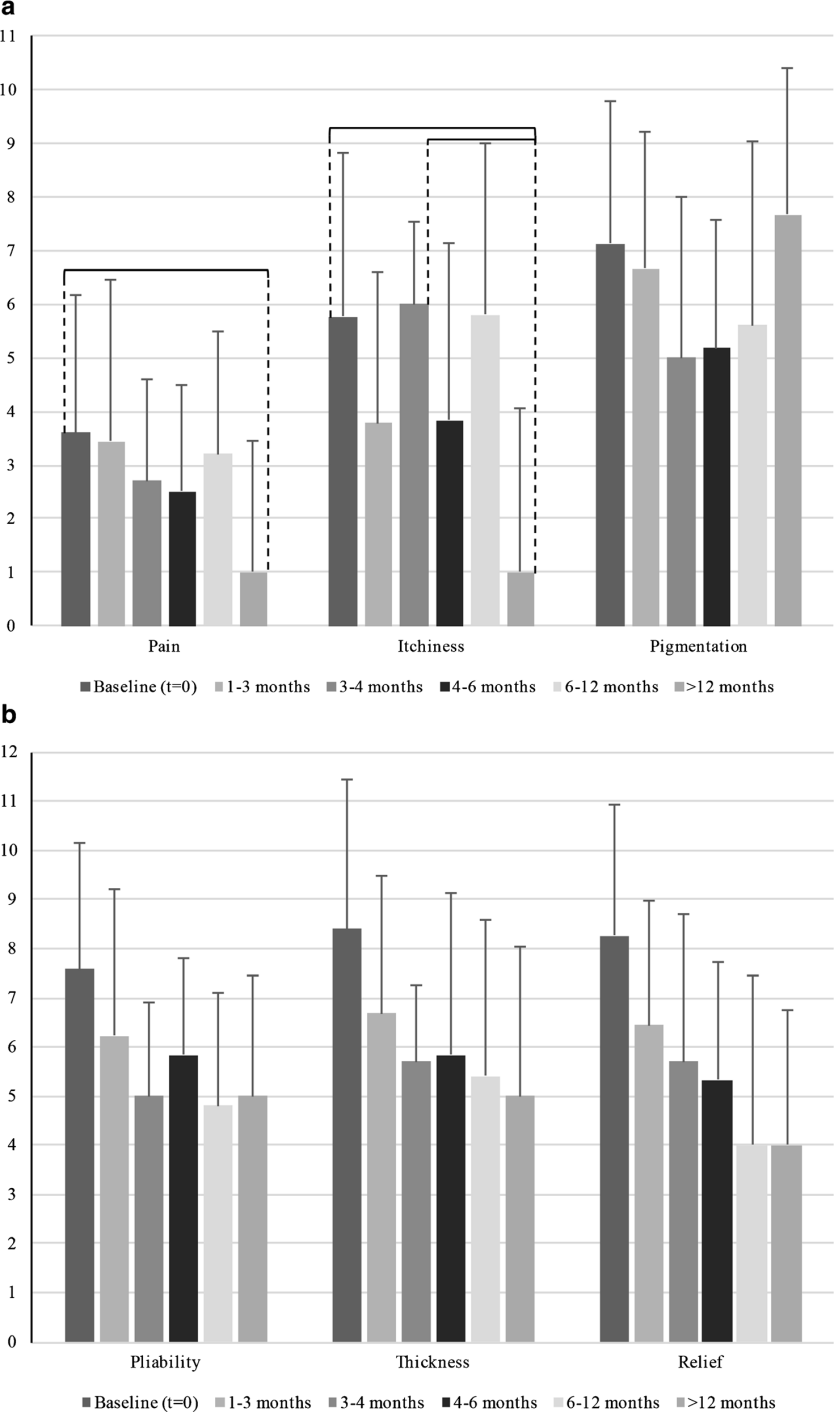
**a** Patient scar scores as part of the total POSAS score are displayed for keloid scars at baseline and five follow-up moments: 1–3 months, 3–4 months, 4–6 months, 6–12 months, and >12 months. Scars were rated on a ten-step scale. *Braces* indicate a statistical significant (*P* < 0.05) difference between follow-up moments. **b** Patient scar scores as part of the total POSAS score are displayed for keloid scars at baseline and five follow-up moments: 1–3 months, 3–4 months, 4–6 months, 6–12 months, and >12 months. Scars were rated on a ten-step scale. *Braces* indicate a statistical significant (*P* < 0.05) difference between follow-up moments

All six components of the Patient Score decreased after baseline, significant differences were found in pain and itchiness (Fig. 3).

#### Pain

There was significant improvement in pain between baseline (3.60, SD: 2.58) and >12 months (1.00, SD: 2.44).

#### Itchiness

Itchiness showed significant decrease between baseline (5.77, SD: 3.05) and >12 months (1.00, SD: 3.04) and between 3 and 4 months (6.00 SD: 1.55) and >12 months.

### Hypertrophic scars

**Fig. 4:**
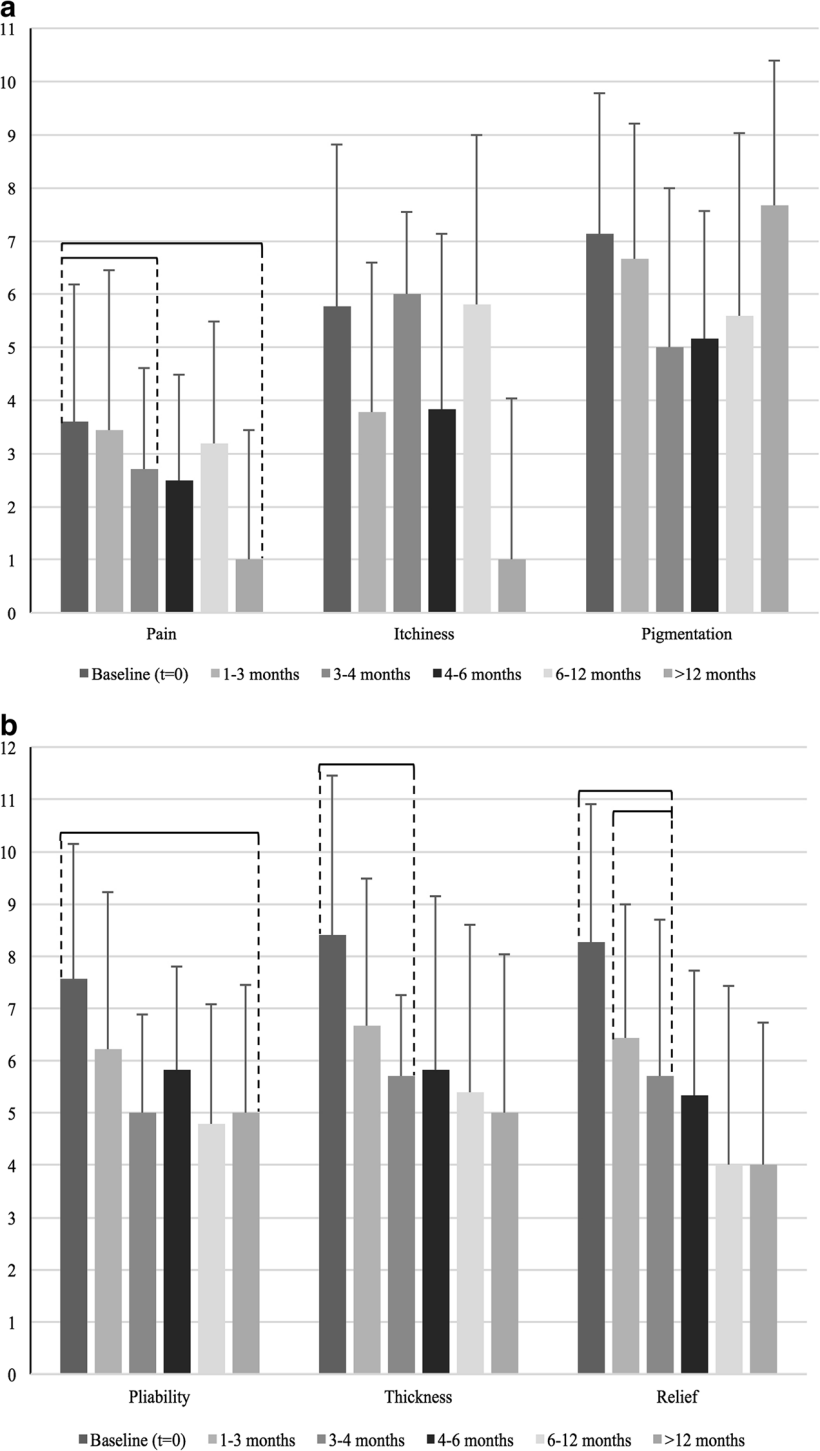
**a** Patient scar scores as part of the total POSAS score are displayed for hypertrophic scars at baseline and five follow-up moments: 1–3 months, 3–4 months, 4–6 months, 6–12 months, and >12 months. Scars were rated on a ten-step scale. *Braces* indicate a statistical significant (*P* < 0.05) difference between follow-up moments. **b**. Patient scar scores as part of the total POSAS score are displayed for hypertrophic scars at baseline and five follow-up moments: 1–3 months, 3–4 months, 4–6 months, 6–12 months, and >12 months. Scars were rated on a ten-step scale. *Braces* indicate a statistical significant (*P* < 0.05) difference between follow-up moments

All of the components of the patient score decreased after baseline, significant differences were observed in pain, scar pliability, thickness, and relief (Fig. 4).

#### Pain

Significant decreases in pain were observed between baseline (3.60, SD: 2.58) and 3–4 months (2.71, SD: 1.89).

#### Pliability

Hypertrophic scar pliability showed significant improvement between baseline (7.57, SD: 2.03) and >12 months (5.00, SD: 2.44).

#### Thickness

There was significant improvement in scar thickness between baseline (8.40, SD: 1.38) and 3–4 months (5.00, SD: 2.41).

#### Relief

Significant decreases were observed between baseline (8.27, SD: 1.86) and 3–4 months (5.71, SD: 2.69) and between 1 and 3 months (6.44, SD: 2.92) and 3–4 months.

### Observer Scores

Corresponding to analyses of Patient Scores all Observer Score components (vascularization, pigmentation, thickness, relief, pliability, and surface area) were compared on baseline and five follow-up moments. A one-way ANOVA with analyses using Games-Howell post-hoc test was used.

### Keloids

**Fig. 5:**
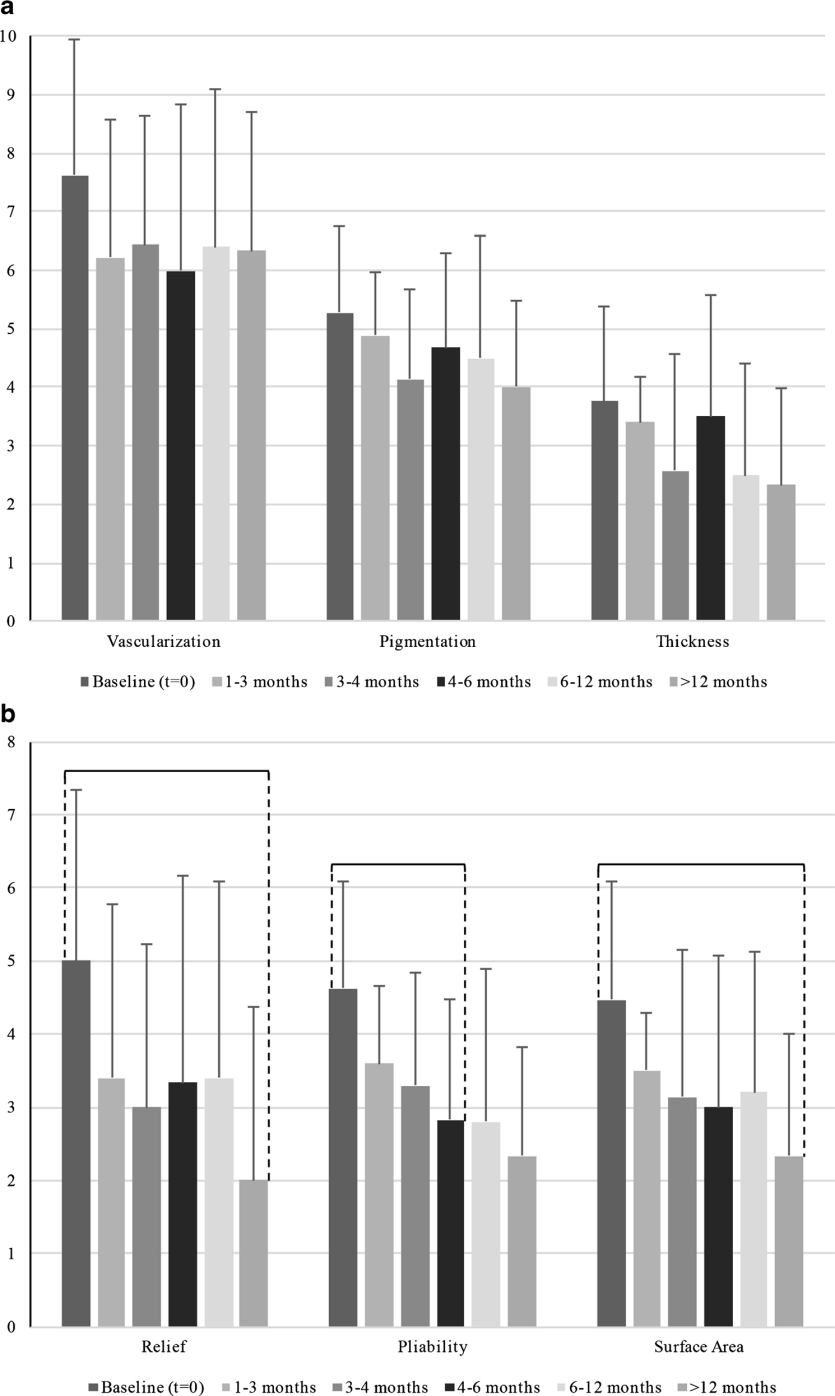
**a** Observer scar scores as part of the total POSAS score are displayed for keloid scars at baseline and five follow-up moments: 1–3 months, 3–4 months, 4–6 months, 6–12 months, and >12 months. Scars were rated on a ten-step scale. *Braces* indicate a statistical significant (*P* < 0.05) difference between follow-up moments. **b** Observer scar scores as part of the total POSAS score are displayed for keloid scars at baseline and five follow-up moments: 1–3 months, 3–4 months, 4–6 months, 6–12 months, and >12 months. Scars were rated on a ten-step scale. *Braces* indicate a statistical significant (*P* < 0.05) difference between follow-up moments

All six components of the observer score decreased after baseline, statistical significant differences were found in scar relief, pliability, and surface area (Fig. 5).

#### Relief

Scar relief showed significant improvement between baseline (5.00, SD: 1.91) and >12 months (2.00, SD: 1.88).

#### Pliability

Significant improvement in scar pliability was observed between baseline (4.63, SD: 1.75) and 4–6 months (3.33, SD: 1.37).

#### Surface area

Surface area of the scar improved significantly between baseline (4.47, SD: 1.59) and >12 months (2.33, SD: 1.63).

### Hypertrophic scars

Every component of the observer score decreased after baseline, statistical significant differences were found in scar vascularization, pigmentation, relief, pliability, and surface area (Fig. 3).

#### Vascularization

There was significant improvement in vascularization between baseline (7.62, SD: 2.34) and >12 months (6.33, SD: 2.36).

#### Pigmentation

Scar pigmentation showed significant improvement between baseline (5.28, SD: 1.46) and 6–12 months (4.50, SD: 2.08) and between 1 and 3 months (4.89, SD: 1.07) and 6–12 months.

#### Relief

Significant decreases in scar relief were observed between baseline (5.00, SD: 1.91) and 4–6 months (3.33, SD: 1.37), 6–12 months (3.40, SD: 0.89) and >12 months (2.00, SD: 1.88).

#### Pliability

There were significant differences in pliability between baseline (4.63, SD: 1.75) and 6–12 months (2.80, SD: 1.30) and >12 months (2.33, SD: 1.78).

#### Surface area

Surface area of the scar showed significant improvement between baseline (4.47, SD: 1.59) and 1–3 months (3.50, SD: 1.57) and 6–12 months (3.20, SD: 1.30).

Summarizing, all POSAS scar aspects showed a decrease in scar score at some moment during follow-up visits, whereas pain, itchiness, pliability, relief, and scar surface area decreased statistically significant (*P* < 0.05) for keloids. For hypertrophic scars, significant decreases in POSAS scores were observed for pain, pliability, thickness, relief, vascularization, pigmentation, and surface area.

### Strengths and limitations

This is the first clinical case-series to evaluate the effectiveness of an intralesional combination therapy for scars with triamcinolone and verapamil. There were several limitations of this study. The number of patients at each follow-up visit would preferably have been larger. Another limitation is the absence of a control group. However, a clear decrease in POSAS scores at all follow-up moments compared to baseline was observed for both keloids and hypertrophic scars. The strength of this study is that it shows clearly that the patients that underwent a full treatment according to our regimen had a fast improvement of their scars. And this was even seen in scars that were already treated with different types of scar therapy before. In this study, the intralesional injections and scar assessments were always carried out by two separate experts.

Furthermore, we did see that patients followed up longer than 12 months also had a strong decrease in the POSAS score. This proves the effectiveness of the combination of triamcinolone and verapamil for intralesional treatment of hypertrophic scars and keloids in the long term.

## Discussion

In this retrospective study, a combined therapy with triamcinolone and verapamil injections resulted in significant scar improvement over time. A total of 116 POSAS scores were collected to evaluate hypertrophic and keloid scarring over a maximum period of 729 days.

The most notable effects from combined triamcinolone and verapamil injection therapy in scar tissue for keloid scars were improvement in scar surface area, pliability, relief, pain, and itchiness (Figs. 3 and 5).

The most notable effects in hypertrophic scars were improvement in pigmentation, vascularization, pliability, thickness, pain, and surface area.

Particularly improvement in thickness, irregularity and pliability can be seen as valuable progress in thickened hypertrophic and keloid scars with excessive collagen deposits.

This study suggests that the combined verapamil and triamcinolone therapy scheme to cause notable scar improvement in both keloid and hypertrophic scars in a relatively early stage (3 to 4 months after start of therapy) (Table 4, Figs. 1 and 2). Our results suggest a beneficial effect on some of the clinical parameters of the Patient Scale, which is an encouraging observation since keloids and hypertrophic scars can cause significant psychological and functional distress [16, 17].

In keloid scars, the same amount of statistically significant decreases in scar scores over time were observed at the Patient and Observer Scale (3; Figs. 3 and 5). The Observer Scale showed significant decreases in relief, pliability, and scar surface area. Significant decreases observed in Patient Score included pain and itchiness.

**Fig. 6:**
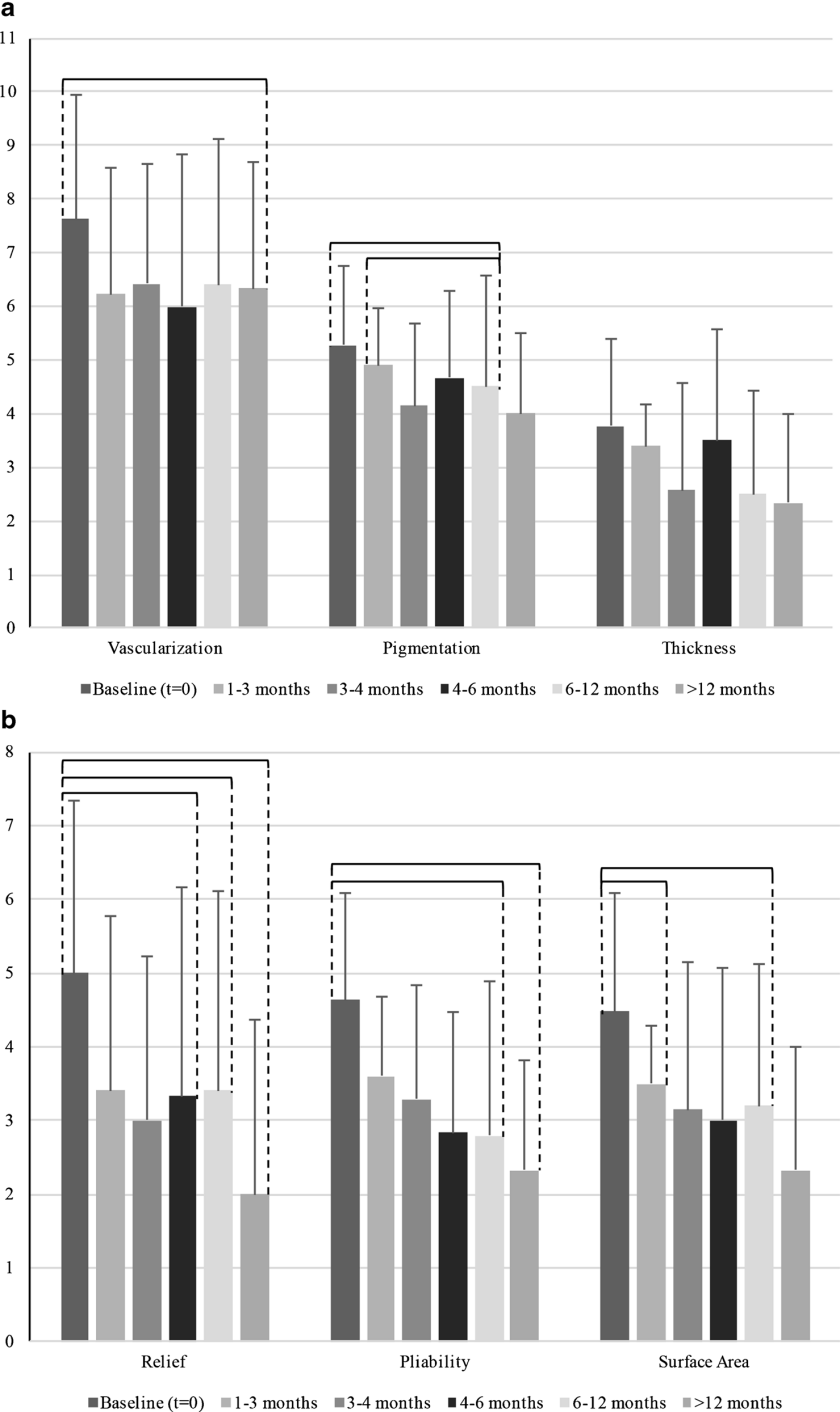
**a** Observer scar scores as part of the total POSAS score are displayed for hypertrophic scars at baseline and five follow-up moments: 1–3 months, 3–4 months, 4–6 months, 6–12 months, and >12 months. Scars were rated on a ten-step scale. *Braces* indicate a statistical significant (*P* < 0.05) difference between follow-up moments. **b**. Observer scar scores as part of the total POSAS score are displayed for hypertrophic scars at baseline and five follow-up moments: 1–3 months, 3–4 months, 4–6 months, 6–12 months, and >12 months. Scars were rated on a ten-step scale. *Braces* indicate a statistical significant (*P* < 0.05) difference between follow-up moments

For hypertrophic scars, the Observer Scale scores show more statistically significant decreases in scar scores over time than the Patient Scale (10 versus 5; Figs. 4 and 6). Every aspect of the Observer Scale demonstrated significant decrease during follow-up, except for thickness. At the Patient Scale, non-significant decreases were observed in scar pigmentation and itchiness.

However, patients’ overall opinions about their abnormal scar are not significantly influenced by itchiness and pigmentation. Instead, psychological distress is suggested to be the more influential characteristic in patients’ overall opinion of their scars [16–18].

Even though POSAS does not include a component of psychological distress or (lack of) quality of life the patient encounters, it is encouraging to see that Patient Scores (including scar pliability, thickness, and relief) reveal prominent improvements in scarring over time.

Multiple studies have proven the effect of triamcinolone and verapamil separately, whereas triamcinolone still is considered being a gold standard in non-surgical management for hypertrophic scarring and keloids. Nonetheless, verapamil has shown to be a promising extra modality in treatment of keloid and hypertrophic scar and it may even function as a suitable alternative to triamcinolone in the treatment of hypertrophic scars and keloids [19, 20].

In an animal model, intralesional administration of verapamil has proven to suppress proliferation and viability of fibroblasts in mice. Furthermore, combination therapy of triamcinolone and verapamil exerted an efficacy equivalent or even better than double-dose verapamil alone in the treatment of hypertrophic burn scars in mice [21].

Correspondingly, a randomized parallel group study concluded that both triamcinolone and verapamil could achieve scar flattening in hypertrophic scars and keloids, yet it needed to be clinically investigated if both drugs could be combined in a single injection to derive a synergistic and enhanced response [22].

The results of the abovementioned studies confirm and rectify our choice to use combined therapy.

**Fig. 7:**
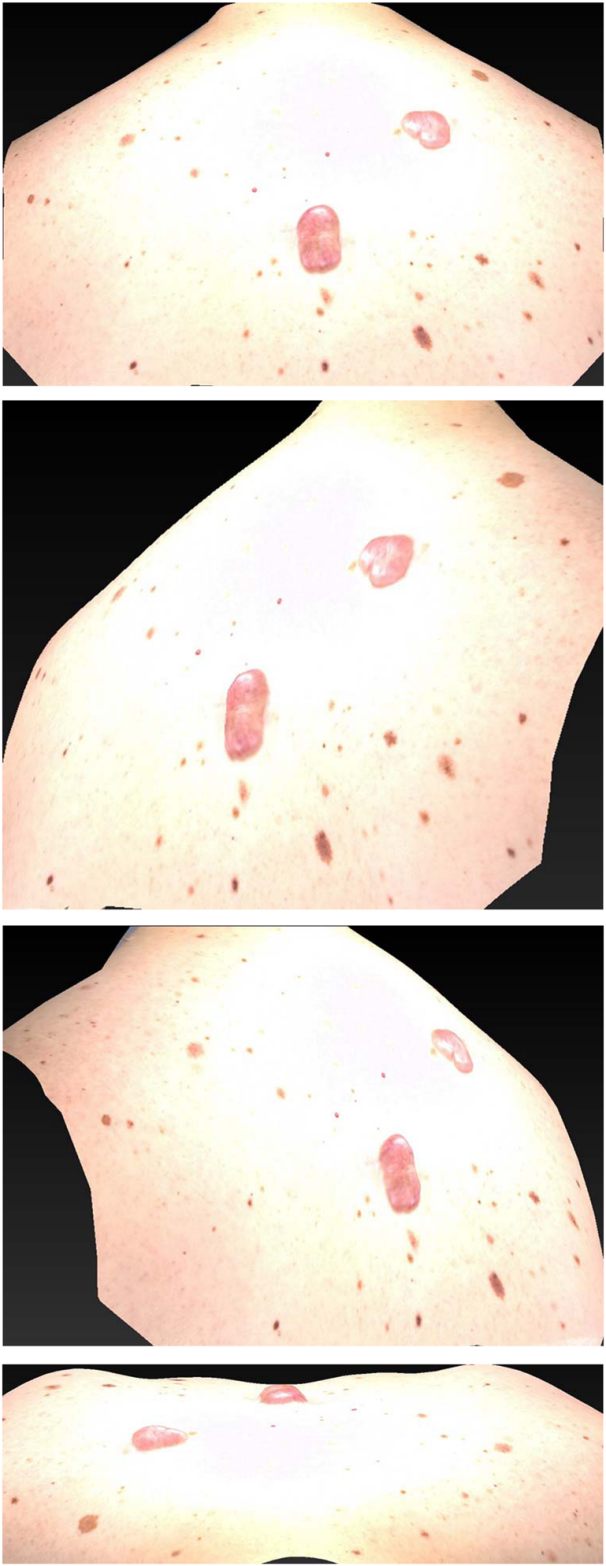
A 42-year-old male patient with multiple keloids at the start of the injection scheme

**Fig. 8:**
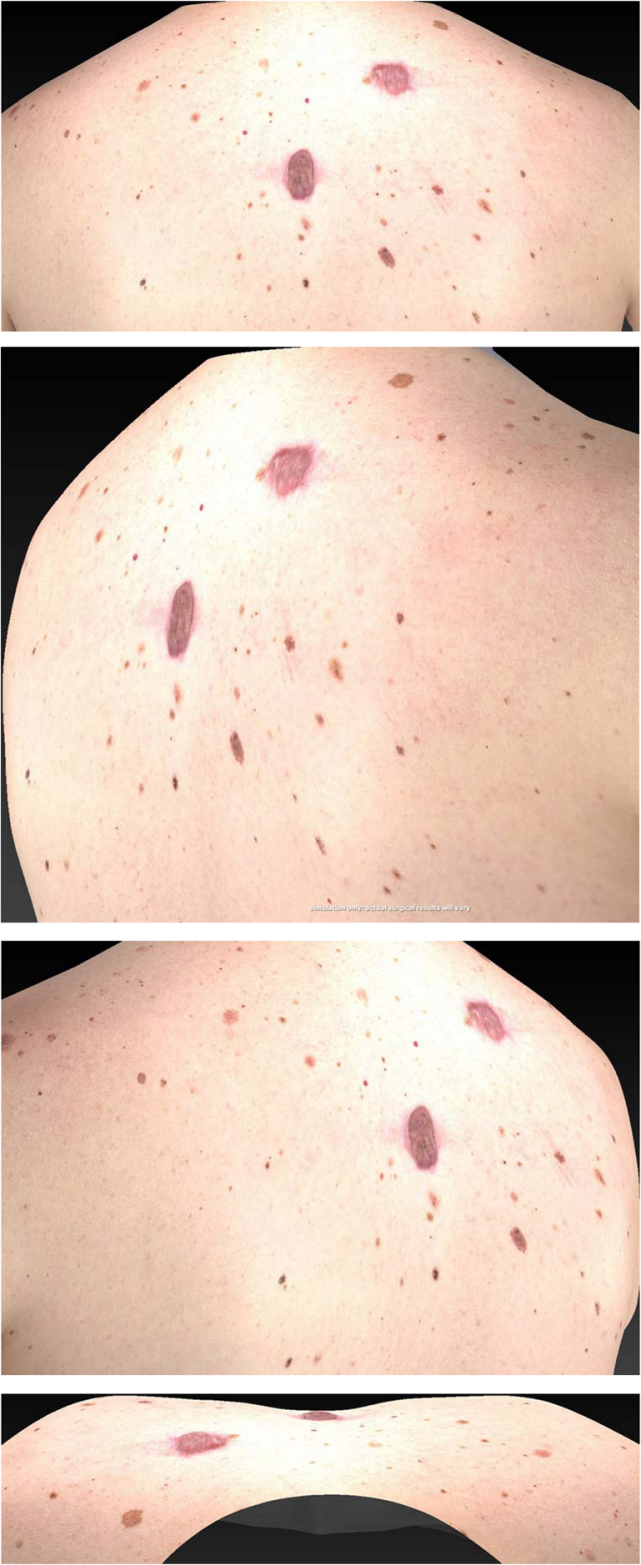
The same 42-year-old male patient after patient completing the full injection scheme 7 weeks later

This study was planned to evaluate the efficacy of triamcinolone and verapamil with respect to scar outcome. According to our results, we assume a combination therapy of triamcinolone and verapamil is a useful modality to treat hypertrophic and keloid scars (Figs. 7 and 8).

This retrospective study showed that a combination therapy of triamcinolone and verapamil results in important scar improvement with a long-lasting result. Future research by means of well-controlled double-blind clinical trials with larger study populations and with the presence of a control group would be ideal for further clinical appraisal of the efficacy of combination therapy of triamcinolone and verapamil.
